# Letter representations in writing: an fMRI adaptation approach

**DOI:** 10.3389/fpsyg.2013.00781

**Published:** 2013-10-28

**Authors:** Olivier Dufor, Brenda Rapp

**Affiliations:** ^1^Electronics department, The Mines-Telecom InstituteTelecom Bretagne, Brest, France; ^2^Department of Cognitive Science, Johns Hopkins UniversityBaltimore, MD, USA

**Keywords:** letter, writing, fMRI, fMRI adaptation, neural habituation, letter shape, letter identity, letter case

## Abstract

Behavioral and neuropsychological research in reading and spelling has provided evidence for the role of the following types of orthographic representations in letter writing: letter shapes, letter case, and abstract letter identities. We report on the results of an fMRI investigation designed to identify the neural substrates of these different representational types. Using an fMRI adaptation paradigm we examined the neural distribution of inhibition and release from inhibition in a letter-writing task in which, on every trial, participants produced three repetitions of the same letter and a fourth letter that was either identical to (no-change trial) or different from the previous three (change trial). Change trials involved a change in the shape, case, and/or identity of the letter. After delineating the general letter writing network by identifying areas that exhibited significant neural adaptation effects on no-change trials, we used deconvolution analysis to examine this network for effects of release from inhibition on change trials. In this way we identified regions specifically associated with the representation of letter shape (in the left SFS and SFG/pre-CG) and letter identity [in the left fusiform gyrus (FG)] or both [right cerebellum, left post-central gyrus (post-CG), and left middle frontal gyrus (MFG)]. No regions were associated with the representation of letter case. This study showcases an investigational approach that allows for the differentiation of the neurotopography of the representational types that are key to our ability to produce written language.

## Introduction

Producing written language requires the recruitment and intricate coordination of a number of processes and representations. While behavioral studies with individuals with neurological damage as well as neurologically intact individuals have provided an increasingly more detailed understanding of the cognitive aspects of these processes and representations, far less is known regarding their specific neural instantiation. In the study we report on here, we investigated the neural substrates of the representation of letter identity, shape, and case via the novel application of an fMRI adaptation paradigm. This approach allowed us to examine the nature of orthographic neural representations in a manner that is considerably more direct than more traditional approaches to these questions.

## The processes and representations of written spelling

The spelling of familiar words is generally assumed to involve the retrieval from long-term memory (orthographic LTM) of the stored representations of word spellings that have been previously learned [for reviews see Roeltgen (1993); and Tainturier and Rapp (2000)]. In alphabetic languages, these lexical orthographic representations include information about letter identity and order, among other things (see Rapp and Fischer-Baum, in press). Letter identity is assumed to be represented in an abstract, modality-independent format in which neither letter-shape nor font are specified, and the representation and retrieval of orthographic lexical representations is assumed to be sensitive to word frequency. In some written codes, in addition to lexical retrieval, word spellings can be assembled sub-lexically based on knowledge of the systematic relationships between sounds and letters (or other orthographic elements). Whether spellings are retrieved from orthographic LTM or assembled via phonology-to-orthography conversion, the representations must be maintained active in orthographic working memory (WM) while each letter/element is serially selected for production, and orthographic WM is assumed to be sensitive to the length of the words held in WM. There is considerable evidence that representations held in orthographic WM are not simply linear letter strings but are, instead, internally structured into syllabic and subsyllabic and more fine-grained structures [for a review see Rapp and Fischer-Baum (in press)]. Subsequently, written spelling requires the transformation of the abstract, format-independent letter identities held in orthographic WM into effector-specific muscle movements. This is a highly complex process involving multiple representational types and processes. For example, in addition to a possible role for a strictly visual representation of letter shape in writing [for discussion, see Menichelli et al. (2008)] a number of motor-based representations have been proposed [see Rapp and Caramazza (1997), for a review]. Among these are shape representations consisting of effector-independent descriptions of the basic strokes (or stroke targets) required to create the intended letter shapes (e.g., *T* = downward vertical + rightward horizontal), sometimes referred to as abstract motor plans (Lashley, 1951; Keele, 1981; Rapp and Caramazza, 1997). Other processes and representations required for the conversion of an abstract motor plan into muscle movements may involve, among other things, specifying information about the sequence, timing, force of strokes, etc., as well as the various spatial coordinate frame transformations required for producing the movements in space.

## Written spelling: neural substrates

There is increasing consensus regarding the basic components of a network of regions that instantiate the operations and representations involved in written spelling that are situated within the broader (and evolutionarily older) neural circuitry that supports visual, language, and motor processing more generally. Two recent meta-analyses of functional neuroimaging studies of word spelling/writing by Purcell et al. (2011b) and Planton et al. (2013) provide excellent characterizations of the current state of understanding of this topic. While there is some overlap between the studies included in each of the meta-analyses, the Purcell et al. meta-analysis is somewhat more weighted toward studies investigating the central and lexical aspects of spelling/writing, while the Planton et al. study is more weighted toward the motor production aspects of spelling/writing. In their most general analyses, both studies identify the following areas: left superior frontal gyrus/sulcus (SFG/SFS; BA6), left pre-central gyrus (pre-CG; BA4), left post-central gyrus (post-CG; BA3), left supplementary motor areas (SMA; BA6), left superior and inferior parietal lobules (SPL; BA7 and IPL; 40), left fusiform/inferior temporal gyrus (FG/ITG; BA 37), right cerebellum and, subcortically, the left thalamus and putamen. For the two studies, the highest activation likelihood estimates—ALE values—(Laird et al., 2005) identified within these neuroanatomical regions were all within 0–13 mm (Euclidean distance) of one another, with the exception of the IPL sites. The major difference in the findings of the two meta-analysis was the identification by Purcell et al. of a left posterior inferior frontal gyrus (IFG) site as well as bilateral superior temporal gyrus/sulcus sites. Both of these differences can be explained on the basis of the aforementioned differences between the two studies in terms of their inclusionary criteria.

Both meta-analyses also attempted to summarize the literature regarding more specific aspects of the processes carried out in the various components of the spelling/writing networks that were identified. They did so by carrying out further analyses on sub-sets of studies according to the types of experimental/control task contrasts that were used in the studies. Although somewhat coarse-grained groupings of studies were (necessarily) used for this purpose, the analyses showed convergence across studies in terms of the association of the fusiform, IFG, STG/STS, and some areas of SPL/IPL with central components of spelling (lexical and WM processes), while the SFS, pre-CG, areas of IPL/SPL, and the right cerebellum were more closely associated with motor planning and programming in writing. It is important to note that these mappings of function to neural substrates are also generally consistent with what has been found from the study of the lesions that give rise to the different types of dysgraphias [see Hillis (2008), for a review]. Further, although these meta-analyses are based on studies of word spelling, there have also been a small number of studies involving single letter writing (Longcamp et al., 2003; James and Gauthier, 2006) that also identified recruitment of left mid/posterior fusiform and left precentral areas in single letter writing compared to typing or picture drawing.

As the meta-analyses indicate, most neuroimaging studies of written word production have used comparisons between experimental and control tasks in order to further our understanding of the specific processes instantiated in the various components of the writing network. Another approach was taken by Rapp and Dufor (2011) who used an fMRI parametric variation approach with a written spelling to dictation task. Rather than comparing experimental vs. control tasks, the logic of this approach is to compare neural responses across experimental conditions that vary in levels along one representational dimension of interest but are matched along others. The expectation is that neural substrates encoding a particular representational dimension should be sensitive to levels of variation along the manipulated dimension but not others. Rapp and Dufor (2011) specifically investigated the distribution of brain areas that were sensitive to lexical frequency but not word length (measured in terms of number of letters), and vice versa. A differential response to frequency level (low vs. high) was assumed to be a marker of lexical orthographic processes, while a differential response to length levels (long vs. short) was assumed to be a marker of orthographic WM processes. Using this approach they identified lexical frequency-sensitive areas in the left IFG (specifically the inferior frontal junction—IFJ), the left fusiform, anterior cingulate, and the thalamus and putamen while length-sensitive areas were identified in the left SFS and SPL.

## fMRI adaptation

This summary reveals that task-comparison and parametric approaches have been used profitably to inform our understanding of the neural substrates of written language production. Another approach that has been used in other cognitive domains is fMRI adaptation. This approach is based on the understanding that when neurons are repeatedly stimulated, their responses decrease in magnitude, possibly because of habituation or lowered thresholds, although the precise nature of the mechanism/s responsible for the response decrease is debated (Grill-Spector et al., 2006). A decrease in signal with repetition of the same stimulus has been reliably measured using fMRI (Grill-Spector et al., 2001) and this has formed the basis of a powerful method for identifying the representational categories processed in specific brain regions. The approach involves presenting an identical stimulus repeatedly (e.g., the same visual image of a dog) which produces a decreasing adaptation/habituation response in brain regions involved in processing the stimulus. After several repetitions, a test image is presented, either the same image again (no-change trial) or a new image (change trial). In no-change trials, activation is expected to decrease further; change trials, on the other hand, because they may allow for a release from inhibition, provide an opportunity to identify the stimulus properties that a particular brain area is sensitive to. For example, the change trial may involve an image of the same dog seen from a different orientation. If a region represents objects in an orientation-invariant manner, then this image would be represented in the same way as the previous ones and one would expect further neural adaptation. However, if the brain area does not generalize across orientation, then the new image may be processed by a different group of neurons and, in that case, a “release from inhibition” and a concomitant increase in activation are expected. In this way, the fMRI adaptation paradigm allows for subtle manipulations of stimulus properties in order to investigate detailed aspects of representation that may be difficult to examine using other approaches. In addition, it provides a means for identifying effects that might be present at a sub-voxel level. This is possible because adaptation and release for inhibition are expected to yield signal differences for a functional voxel even if different sub-populations of neurons within that voxel are involved in representing the different stimulus properties.

In the study we report on here we apply, for the first time, an fMRI adaptation paradigm to written letter production with the goal of investigating the neural representation of letter identity, shape, and case (upper/lower case). The logic is very straightforward: participants are prompted to write three repetitions of a particular letter which is expected to produce adaptation/habituation effects in brain areas involved in letter production. On the fourth trial, either the same letter (No-Change trial) or a different letter (Change trial) is produced. Change trials can involve a change in case only (vvvV), a change in case and shape (dddD), a change in identity and shape (dddg) or a change in identity, case and shape (dddG). Comparisons of the magnitude of activation across the different types of Change trials should reflect the extent of neural habituation or release for inhibition that has occurred, revealing the type/s of representations processed in a given brain region. In this way, the fMRI adaptation approach provides a method for a detailed examination of the neural substrates of different aspects of letter representation.

## Methods

### Participants

Ten neurologically healthy, native English speakers (5 women) participated in this experiment (mean age = 24.7). All were right handed according to the Edinburgh Inventory (Oldfield, 1971) (average score: 80.33) and were paid for their participation.

### Experimental task

**Figure 1:**
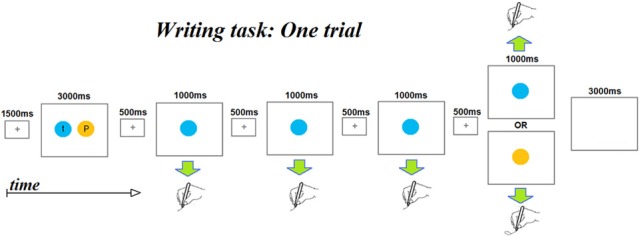
**A schematic depicting the time course of the events that makes up a single fMRI scanner trial**. Each trial begins with a presentation of two colored circles, each containing a letter. Participants had been instructed to encode the color-letter mappings as the colors were to serve as prompts for the letters to be written subsequently within the trial. Note that each trial involves writing four letters. The first three letters are always the same letter, while the fourth may be the same letter (No-change trials) or a different letter (Change trials). Color and letter pairings change from trial to trial.

An fMRI adaption paradigm was used with a letter writing task with the goal of identifying neural substrates sensitive to the representation of letter identity, shape, and/or case. As depicted in Figure 1, each trial consisted of the following sequence of events: (1) a 1500 ms centrally displayed fixation cross; (2) an “instruction” display presented for 3000 ms consisting of a pair of letters, each presented in a circle of a different color. Participants had been instructed to encode the association between each color and letter during this display. (3) A series of four displays in which only one of the two colored circles from the “instruction display” was centrally presented. Each display had a duration of 1500 ms, consisting of a 500 ms central fixation cross and a 1000 ms colored circle. Participants had been instructed that each time a colored circle appeared, they were to quickly write the letter associated with the color. The first three displays of a series always presented the same color while the fourth displayed either the same color as the preceding three (referred to as a “No Change” trial) or displayed the other color (referred to as a “Change” trial). (4) A 3000 ms inter-trial interval with a blank screen. The total duration of each trial was 13.5 s. Five colors (red, blue, yellow, green, and purple) were used and associations between colors and letters were counterbalanced so that each letter was associated with every color. A notepad was attached with Velcro to the participant's thigh to enable supine writing without extraneous movements; participants could not see what they were writing and were asked to write letters on top of one other to minimize arm movements.

Stimuli consisted of upper and lower case versions of 8 different 2-stroke letters (dgpqstvx/DGPQSTVX). Letter pairs were selected to allow for letter identity (I), shape (S), and/or case (C) to vary across the letters in a pair. There were four relationships between the letter pairs: (1) Case (C) involved a difference in case only (maintaining letter shape and identity): sS, vV, xX, pP. (2) Case/Shape (CS) involved a difference in case and shape (maintaining letter identity): qQ, tT, gG, dD. (3) Identity/Shape (IS) involved a difference in identity and shape (maintaining case): xp, sv, SP, VX. (4) Identity/Case/Shape (ICS) involved a difference in all three dimensions: gT tQ, dG, qD. Within-trial letter presentation order was counter balanced.

These four stimulus types allowed us to examine neural sensitivity to each of the three dimensions of letter representation by comparing the activation magnitudes for all the trial types involving one type of change vs. those that did not involve that change. As can be seen in Table 1, trial types C, CS, and ICS all involve a change of case whereas the IS trial type does not. Regions representing letter case should exhibit a greater release from inhibition (higher activation) for the case-change trials (e.g., sssS, dddD, dddG) compared to trials on which case is maintained (e.g., dddg). By the same logic, regions encoding letter identity should exhibit higher activation when letter identity is changed (**I**CS + **I**S) compared to when it is not (CS + C) and, finally, sensitivity to shape should result in stronger activity for trials that involve a shape change (IC**S** + I**S** + C**S**) compared to those that do not (C). There are several reasons why trial types were grouped for these analyses. One is that this helped address the shortcoming created by the relatively small number of trials per type. More importantly, grouping was necessary because identity changes are necessarily accompanied by shape changes and, similarly, changes in shape are necessarily accompanied by changes in identity or case. As a result there was no single contrast that can be used to isolate identity or shape. However, the grouping of trial types (e.g., **I**CS + **I**S vs. CS + C) allowed this issue to be addressed.

**Table 1:**
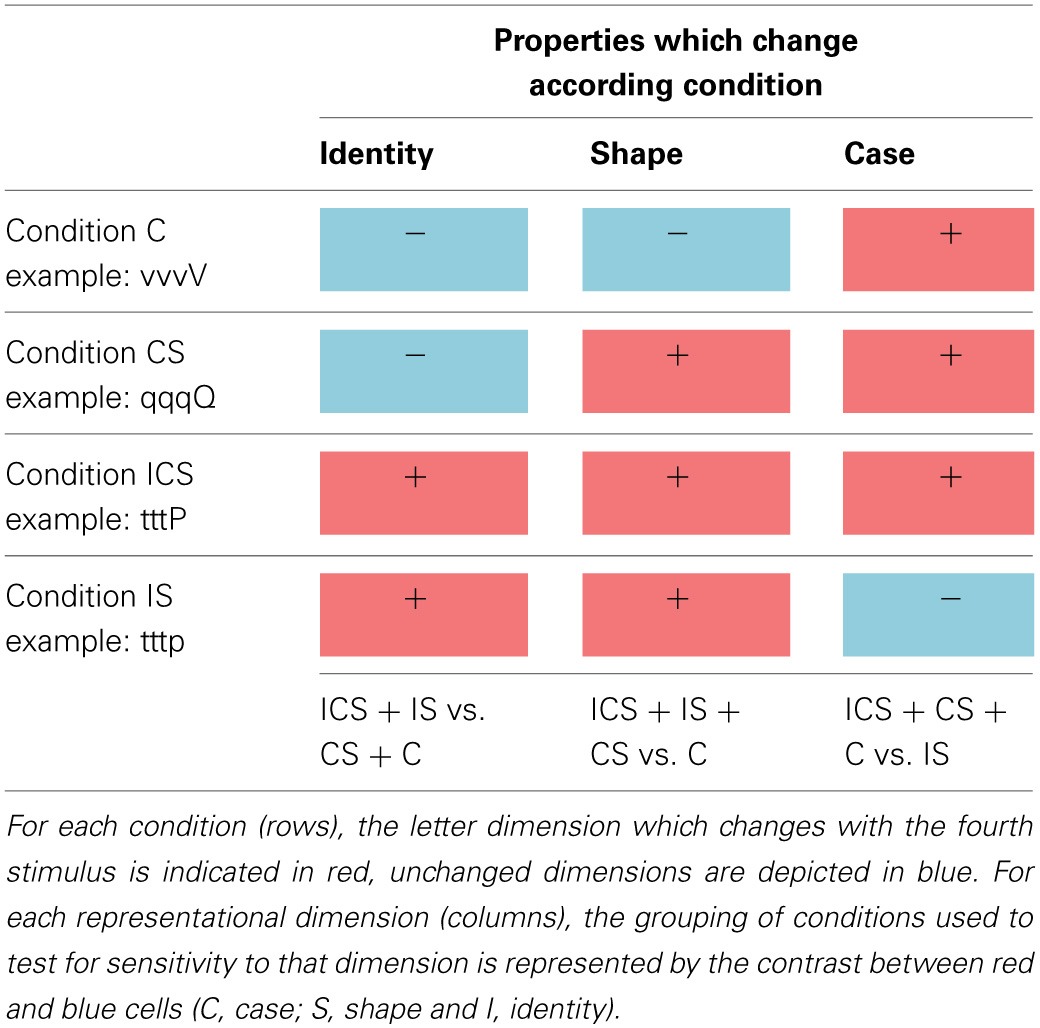
**The four experimental stimulus conditions (C, CS, IS, and ICS) used in Change trials are depicted in the rows, and each representational dimension investigated (identity, shape, and case) are depicted in the columns**.

For each condition (rows), the letter dimension which changes with the fourth stimulus is indicated in red, unchanged dimensions are depicted in blue. For each representational dimension (columns), the grouping of conditions used to test for sensitivity to that dimension is represented by the contrast between red and blue cells (C, case; S, shape and I, identity).

Each of the four letter pairs per stimulus type was presented six times (half of the time in one left/right order and the other half in the other) yielding a total of 24 trials per stimulus type. Four of the six displays for each letter pair involved Change trials (total *n* = 16 per stimulus type) and the other two were used in No-Change trials (total *n* = 8 per stimulus). In this way, each subject contributed 16 Change trials to the analysis of release from inhibition for each of the four Change types. In addition, each subject contributed 32 No-Change trials to the analysis of adaptation/habituation effects. Furthermore, there were 12 filler trials involving different letter pairs bringing the total number of trials per subject to 108. The filler trials were originally intended for an entirely different contrast and were structured just like the other trial types but, because the stimuli were not appropriately matched, they were not subjected to analysis, Effectively there were a total of 6 experimental conditions: 4 corresponding to each of the Change trials for each of the 4 stimulus types, 1 corresponding to all of the No-Change trial combining the 4 stimulus types and 1 corresponding to the filler trials. The 108 trials were distributed in 4 experimental scanning runs (*n* = 27 trials/run) such that trial types were matched across runs and randomly presented within each run yielding 251 volumes per run (27 trials × 9 volumes/trial + 2 × 4 volumes of blank screen at the beginning and end of each run). Stimuli were presented using E-Prime 2.0 software (Psychology Software Tools, Pittsburgh, PA), Schneider (Schneider et al., 2002a,b).

Prior to the scanning sessions, all participants underwent a practice session in which they were familiarized with the task until they could carry it out easily and accurately.

### Imaging parameters

MRI data were acquired with a 3.0-T Phillips Intera Scanner at the F. M. Kirby Research Center for Functional Brain Imaging at the Kennedy Krieger Institute (Baltimore, MD). Whole-brain T2-weighted gradient-echo, EPIs were acquired with an eight-channel SENSE (Invivio) parallel-imaging head coil in 29 transverse slices (*TR* = 1500 ms, echo time = 30 ms, flip angle = 65°, field of view = 240 × 240 mm, matrix 128 × 128, slice thickness = 4 mm, gap = 1 mm). Structural images were acquired using an MP-RAGE T1-weighted sequence that yielded images with a 1-mm isotropic voxel resolution (*TR* = 8.06 ms, echo time = 3.8 ms, flip angle = 8°).

### Data analysis

All analyses were carried out using Brain Voyager QX [1.10.4] (Brain Innovation, Maastricht, The Netherlands) (Goebel et al., 2006). In terms of pre-processing, functional images were slice-time corrected using sinc interpolation and a temporal high-pass filter was applied to remove components occurring fewer than three cycles over the course of a run. For each participant, EPI images were realigned to the first volume according a trilinear sinc interpolation (default) and then motion corrected. The first volume of each subject was coregistered with the anatomical T1 image and the T1 images were then normalized to Talairach space (Talairach and Tournoux, 1988) and resampled to 3-mm isotropic voxels. The normalization step to Talairach template was done by first automatically locating the landmarks: AC, PC, and then manually adjusting them after locating the other landmarks (anterior, posterior, right, and left poles) before applying the transform. A linear expansion or shrinkage of the 12 cuboïds forming the Talairach template. Throughout, locations are reported in Talairach coordinates unless otherwise indicated (Talairach and Tournoux, 1988; Lancaster et al., 1997, 2000). Conversion to MNI coordinates was carried out using a tal2mni.m Matlab script.

We carried out a two-stage analysis, a first stage in which we identified brain regions generally sensitive to letter representation and a second stage in which we examined the identified regions specifically for the representation of letter identity, shape, and/or case. Both analyses were based on a general linear model used to estimate parameter values in an event-related design (Friston et al., 1995). For Analysis 1, the model contained 9 experimental regressors of interest: one for the combined fixation and instruction period of every trial, three regressors for each of the first three stimuli and their respective responses, five regressors for each of the five experimental conditions (No-Change, C Change, IS Change, CS Change, ICS Change) that corresponded to the time period that included the fourth stimulus and response as well as the inter-trial interval. Also included were 10 regressors for the filler trials, structured similarly to the experimental regressors, six motion regressors, and a confound regressor indicating run number were also included. For Analysis 1, time points corresponding to a blank screen at the beginning (6 s) and end of each run (6 s) were left unmodeled and served as the implicit baseline. For Analysis 2, the same GLM was used except that a “beginning and end” regressor was specified and it was the No-Change time points which were left unmodeled and used as the implicit baseline. In Analysis 1, a standard hemodynamic response function (2 gamma functions) was used to model the hemodynamic response. For Analysis 2, a deconvolution approach (Brain Voyager, QX [1.10.4]; Brain Innovation, Maastricht, The Netherlands) was used to estimate the beta values for the 12 time points (with a *TR* = 1500 ms = 18 s) following the onset of each of the 9 experimental regressors. Briefly, the BVQX deconvolution analysis modeled each of the 12 time points using a series of impulse response functions (stick functions), yielding beta values corresponding to each of the time points.

#### Analysis 1: identifying letter-sensitive cortex

The objective of this analysis was to identify neural regions involved in letter representation and processing by identifying those areas that showed neural adaptation/habituation effects in letter writing. To do so, a whole-brain analysis was carried out examining No-Change trials only and contrasting the neural response for the first three repetitions of a letter with the neural response for the fourth (No-Change) repetition. Voxels exhibiting a significant decrease in activation from the first three repetitions to the fourth repetition were identified (contrast = 1 1 1 −3), applying an FDR correction for multiple comparisons (*p* < 0.01) and a minimum cluster threshold of 5 functional voxels.

#### Analysis 2: localizing the dimensions of letter representation-identity, case, and shape

A Volumes of Interest (VOI) analysis was carried out to explore the functional properties of the clusters identified in Analysis 1. Specifically, the goal was to determine if any of these clusters was specifically sensitive to letter identity, shape, and/or case by examining the activation magnitude on Change trials for the voxels within the VOIs identified in Analysis 1. That is, this analysis compared the magnitude of the “release from inhibition” of letter-sensitive voxels for each of the four Change Conditions. To do so, the beta values corresponding to only the first 6 time points (a 9 s time period) of the fourth stimulus of Change trials were used, as this time period effectively captured the neural response.

The beta weights for the first 6 time points of the fourth trials corresponding to each of the four Change conditions (for each of the 10 participants) were evaluated by means of a 3-Way mixed-effects analysis of variance (ANOVA). The following statistical model was used: *Y_ijk_* = μ + α_*i*_ + δ_*j*_ + τ_*k*_ + ε_*ijk*_; where *Y_ijk_* is the response due to subject *i* (*i* = 1 − 10) for condition *j* (*j* = 1 − 4) at Time *k* (*k* = 1 − 6); μis the overall mean of the response; α_*i*_ is a random effect due to subject *i* with α_*i*_ ≈ *N*(0, σ^2^_α_); δ_*j*_ is a fixed effect due to condition *j*; τ_*k*_ a fixed effect due to time *k* with $\sum_{k=1}^{6}\tau_{k}=0$; ε_*ijk*_ is the random error with ε_*ijk*_ ≈ *N*(0,σ^2^_ε_); note that ε_*ijk*_ and α_*i*_ are independent. An interaction term between condition and time was shown to be non-significant and was consequently omitted from the final model based on the Akaïke criteria (AIC) (Akaike, 1974). Model parameters were estimated using the Proc Mixed SAS procedure (SAS/STAT version 9.2, SAS Institute, Inc. Cary, NC).

Predicted marginal beta value means (Least-Square Means) were estimated for the four conditions (the effects of interest). As the experimental design was balanced, these simply correspond to the arithmetic mean of the responses observed within each group. *Post-hoc* pairwise Student tests, adjusted for multiple comparisons using the Scheffe correction, were then implemented and linear contrasts were tested for significance for comparisons of selected groups of conditions. Specifically, three linear contrasts were carried out to test for the following effects: Identity: **I**CS + **I**S *vs*. CS + C, Shape: IC**S** + I**S** + C**S** *vs*. C, and Case: I**CS** + **C**S + **C** vs. IS. Finally, to evaluate whether or not there were significant differences between conditions *within* each group of conditions, pairwise comparisons of conditions applying the Scheffe correction were also carried out.

## Results

### Analysis 1: identifying letter-sensitive cortex

**Figure 2:**
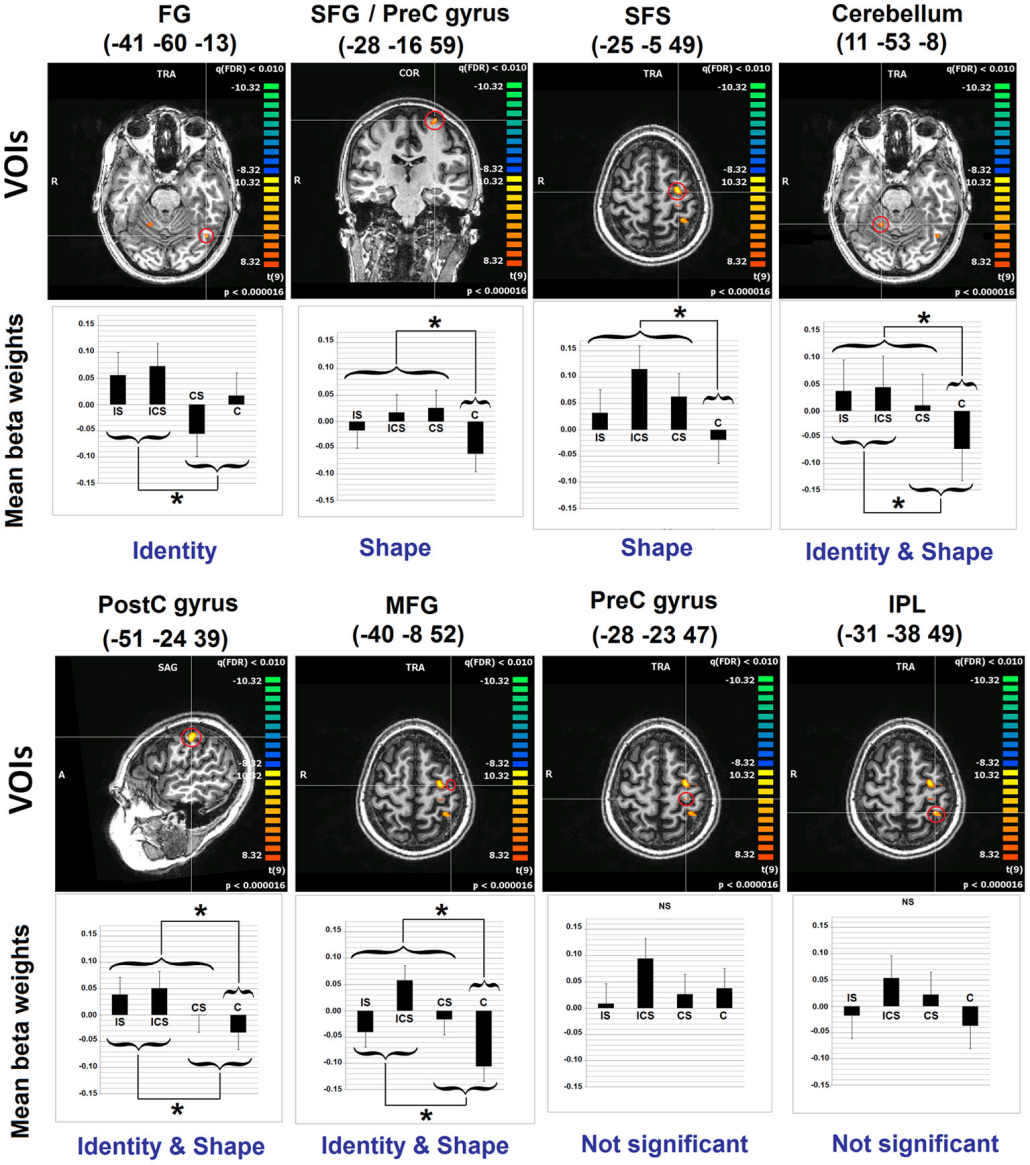
**Results of Analyses 1 and 2**. The 8 clusters identified in Analysis 1 are depicted. With regard to Analysis 2, bar graphs represent the mean beta values (and SEs) across voxels in each of the depicted VOIs, for each of the experimental conditions. Indicated also are the results of the linear contrasts performed at each VOI, with brackets indicating the grouping of conditions that were significantly different from one another (^*^significant at Scheffe corrected *p* < 0.05). The terms Identity and Shape indicate the dimension/s of letter representation that was/were shown, for each VOI, to be significant. See Table 2 for an explanation of the abbreviations for the neuroanatomical terms. VOI, volume of interest; C, case; S, shape; I, identity; SE, standard error.

A whole-brain analysis examining No-Change trials identified voxels exhibiting significant neural adaptation as indexed by a decrease in response from the first three repetitions of a letter to the fourth repetition of the same letter (FDR *p* < 0.01 and *k* 5). Eight clusters were identified and these are depicted in Figure 2, with information regarding the location of the peak voxels and the cluster volumes presented in Table 2. These eight clusters were located as follows: left fusiform gyrus (FG) (BA37: −41, −60, −13), left superior frontal gyrus/Pre-central gyrus (SFG/Pre-CG) (BA6: −28, −16, 59), left superior frontal sulcus (SFS) (BA6: −25, −5, 49), the right cerebellum (Culmen: 11, −53, −8), left middle frontal gyrus (MFG) (BA6: −40, −8, 52), left pre-central gyrus (Pre-CG) (BA4: −28, −23, 47), left post-central gyrus (Post-CG) (BA2: −51, −24, 39), and left inferior parietal lobule (IPL) (BA5/40: −31, −38, 49).

**Table 2:** **Results of Analyses 1 and 2**.

	**Cluster size *k***	**Brodmann area**	**X Tal (MNI)**	**Y Tal (MNI)**	**Z Tal (MNI)**	**Identity effect, linear contrast IS + ICS vs CS + C (and *F* values)**	**Shape effect, linear contrast IS + ICS + CS vs. C (*p* and *F*-values)**	**Case effect, linear contrast IS vs. ICS + CS + C (*p* and *F*-values)**
FG	47	BA37	−41 (−43)	−60 (−59)	−13 (−21)	0.0055^*^ 7.87	0.8296 0.05	0.1993 1.66
SFS	181	BA6	−25 (−25)	−5 (−8)	49 (54)	0.0779 3.14	0.0089^*^ 6.96	0.5423 0.37
SFG/Pre-CG	86	BA6	−28 (−28)	−16 (−20)	59 (66)	0.4887 0.48	0.0191^*^ 5.57	0.7147 0.13
MFG	11	BA6	−40 (−40)	−8 (−11)	52 (57)	0.0105^*^ 6.66	0.0008^*^ 11.55	0.5368 0.38
Post-CG	228	BA2	−51 (−52)	−24 (−26)	39 (42)	0.0248^*^ 5.11	0.0465^*^ 4.01	0.2922 1.11
Cerebellum	49	Culmen	11 (11)	−53 (−53)	−8 (−15)	0.0083^*^ 7.09	0.0011^*^ 10.89	0.1672 1.92
Pre-CG	23	BA4	−28 (−28)	−23 (−26)	47 (51)	0.5550 0.35	0.8837 0.02	0.2534 1.31
IPL	96	BA40	−31 (−31)	−38 (−41)	49 (54)	0.3930 0.73	0.0984 2.75	0.3687 0.81

The eight significant clusters identified in Analysis 1 are listed (FDR corrected p < 0.01), along with the cluster size (k), the Brodmann area and the xyz coordinates (both TAL and MNI) of the peak voxel in each cluster. For Analysis 2, listed are the Scheffe corrected p-values and F-values for the linear contrast VOI analyses performed on each cluster (

* significant at Scheffe corrected p < 0.05). (FF, fusiform gyrus; SFG, superior frontal gyrus; pre-CG, pre-central gyrus; SFS, superior frontal sulcus; MFG, middle frontal gyrus; post-CG, post-central gyrus; IPL, inferior parietal lobule; TAL, Talairach; MNI, Montreal Neurological Institute; S, shape; C, case; I, identity).

### Analysis 2: localizing the dimensions of letter representation-identity, case, and shape

For each of the letter-sensitive regions identified in Analysis 1, the beta values corresponding to the first 6 time points subsequent to the fourth stimulus on Change trials were used to compare the magnitude of the “release from inhibition” across the different Change conditions.

Although, as described earlier, the planned and primary analysis of these data involves the linear contrast of groups of conditions, the individual conditions within each VOI were also evaluated against the null hypothesis with the results reported in Table 3 (means beta values, SE's, *t*-values, and their corresponding *p*-values). Bonferroni correction for multiple comparisons was applied, after which there was only one significant result, namely the finding that activation for Case in the MFG VOI was significantly below baseline. Somewhat puzzling is why activation for any of the Change conditions should be significantly below the baseline, given that baseline represents No Change activation levels. In other words, if there was no release from inhibition—as would be expected if an area were not sensitive to Case—then activation levels comparable to the No Change condition would be expected. Below-baseline activation levels indicate even larger adaptation effects for Case (e.g., vvvV) than for No Change trials in this region. While the interpretation of this effect is unclear, what is clear is that the finding is not consistent with the representation of letter case in this region. The linear contrasts, which were specifically designed to examine the groupings of conditions in order to identify name, shape or case sensitive regions should provide a clearer picture.

**Table 3:** **For each of the 8 VOIs identified in Analysis 1, the mean beta values and SEs (in parentheses) across voxels for each of the experimental conditions are reported**.

	**FG**	**SFG/Pre-CG**	**SFS**	**Pre-CG**	**Cerebellum**	**MFG**	**Post-CG**	**IPL**
IS	0.056 (0.043)	−0.017 (0.034)	0.032 (0.044)	0.009 (0.038)	0.038 (0.060)	−0.041 (0.028)	0.039 (0.032)	−0.018 (0.043)
	*t* = 1.30; *P* = 0.195	*t* = −0.49; *P* = 0.622	*t* = 0.73; *P* = 0.466	*t* = 0.24; *P* = 0.811	*t* = 0.64; *P* = 0.524	*t* = −1.45; *P* = 0.150	*t* = 1.21; *P* = 0.229	*t* = −0.42; *P* = 0.676
ICS	0.0731 (0.043)	0.0174 (0.034)	0.115 (0.044)	0.095 (0.038)	0.045 (0.060)	0.058 (0.028)	0.051 (0.032)	0.054 (0.043)
	*t* = 1.70; *P* = 0.090	*t* = 0.52; *P* = 0.606	*t* = 2.60; *P* = 0.010	*t* = 2.51; *P* = 0.013	*t* = 0.76; *P* = 0.449	*t* = 2.07; *P* = 0.040	*t* = 1.57; *P* = 0.117	*t* = 1.27; *P* = 0.206
CS	−0.055 (0.043)	0.026 (0.034)	0.063 (0.044)	0.027 (0.038)	0.011 (0.060)	−0.016 (0.028)	−0.0004 (0.039)	0.022 (0.043)
	*t* = −1.29; *P* = 0.197	*t* = 0.78; *P* = 0.438	*t* = 1.42; *P* = 0.157	*t* = 0.70; *P* = 0.482	*t* = 0.18; *P* = 0.858	*t* = −0.58; *P* = 0.560	*t* = −0.01; *P* = 0.991	*t* = 0.53; *P* = 0.595
C	0.017 (0.043)	−0.061 (0.034)	−0.019 (0.044)	0.038 (0.038)	−0.072 (0.060)	−0.106 (0.028)	−0.033 (0.032)	−0.037 (0.043)
	*t* = 0.40; *P* = 0.692	*t* = −1.81; *P* = 0.072	*t* = −0.43; *P* = 0.668	*t* = 1.00; *P* = 0.317	*t* = −1.21; *P* = 0.226	*t* = −3.76^*^; *P* = 0.0002	*t* = −1.03; *P* = 0.303	*t* = −0.88; *P* = 0.382

These values correspond to the data plotted in the bar graph in Figure 2. Also included are the results of one sample t-tests for each mean beta value against baseline (t- and p-values);

* significant at Bonferroni corrected p < 0.05.

Inspection of the results of the linear contrasts reveals that the only cluster to exhibit selective sensitivity to Letter Identity (the contrast **I**S + **I**CS vs. CS + C) was in the left FG (−41, −60, −13; *p* = 0.0055). Two regions exhibited selective sensitivity to Letter Shape (the contrast IC**S** + I**S** + C**S** *vs*. C): the superior frontal sulcus (SFS: −25, −5, 49; *p* = 0.0089) and the posterior superior frontal gyrus/pre-central gyrus (SFG/pre-CG: −28, −16, 59; *p* = 0.0191). Selective sensitivity to Letter Case (the contrast I**CS** + **C**S + **C** vs. IS) was not observed in any of the VOIs. However, there were three regions that showed significant effects of both Identity and Shape: the right cerebellum (11, −53, −8; *p*_identity_ = 0.0083; *p*_shape_ = 0.0011); the left post-central gyrus, (Post-CG: −51, −24, 39; *p*_identity_ = 0.0248; *p*_shape_ = 0.0465) and the left middle frontal gyrus, (MFG: −40, −8, 52; *p*_identity_ = 0.0105; *p*_shape_ = 0.0008). Finally, it is worth noting that two clusters that exhibited significant neural adaptation effects as identified in Analysis 1, did not display significant sensitivity to any of the three representational dimensions examined; these were the clusters in the left inferior parietal lobule (IPL) and the left pre-central gyrus (Pre-CG).

Because the contrasts involved comparisons between groups of conditions, it is useful to consider—for each of the significant findings—whether or not there were significant differences amongst the conditions that were grouped together. The results of this *post-hoc* testing with Scheffé adjusted *p*-values are reported in Table 4. Cells shaded in gray are those corresponding to conditions that formed part of the same group in the linear contrast/s that were shown to be significant for that region. Ideally, none of comparisons in these cells should be significant. Consider, for example, the finding that the identity contrast (**I**S + **I**CS vs. CS + C) was significant for the left fusiform cluster. The results reported in Table 4 indicate that IS and ICS conditions did not differ from one another, and neither did CS vs. C, thus providing support for the linear contrast grouping result. Overall, these results indicate no significant within-group differences that would weaken the interpretation of the linear contrast results.

**Table 4:**
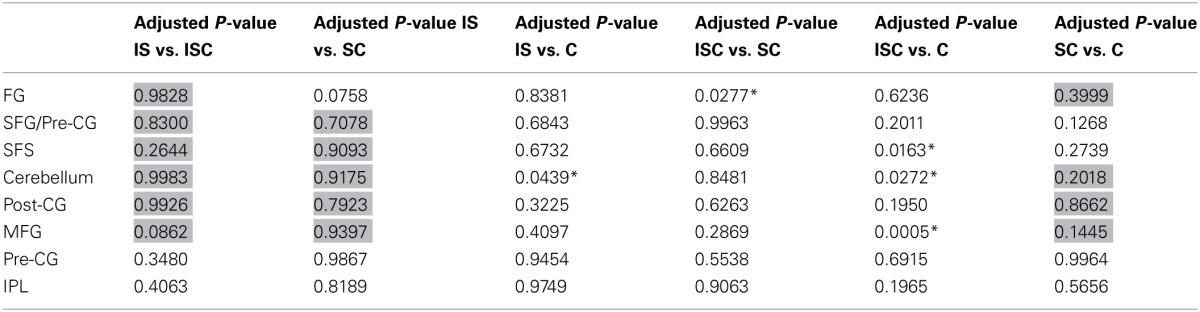
**Scheffé corrected *p*-values for the pairwise comparisons of all experimental conditions for each VOI**.

For each VOI, cells shaded in gray correspond to conditions that form part of the same group in the particular linear contrast/s that was/were shown to be significant in Analysis 2. Non-significant results in the shaded cells support the grouping of these conditions in the analysis. ^*^Significant at Scheffe corrected p < 0.05.

## General discussion

### Using fMRI adaptation to identify the spelling/writing network

Neural habituation effects were used in Analysis 1 to identify neural regions sensitive to the repeated production of the same letter. This approach was designed to capture the broad neural network that supports the numerous cognitive processes involved in letter writing and which can be expected to show habituation/adaptation effects as they are repeatedly engaged. As reported above, this analysis yielded eight significant clusters. Given the novel nature of this approach, it is important to consider the locations of these clusters compared to those identified in the two meta-analyses of spelling/writing carried out by Purcell (Purcell et al., 2011b) and Planton (Planton et al., 2013). For this comparison we consider the results reported by Purcell et al. and Planton et al. based on the complete set of studies they included (rather than additional analyses they reported involving subsets of studies). It is important to note that, in contrast to our investigation, the studies included in the meta-analyses consisted exclusively (for Purcell et al., 2011b) or largely (for Planton et al., 2013) of spelling/writing tasks involving words rather than single letters. Nonetheless, all eight clusters that we identified were located in neuroanatomical regions identified in both meta-analyses. More specifically, six of the eight clusters (fusiform, SFS, PreCG, SFG/Pre-CG, IPL, and cerebellum) were located within 1.1 cm (Euclidean distance) of clusters reported in both studies. One (MFG) was within 1 cm of a cluster reported in Planton et al. and within 2 cm of a cluster reported in Purcell et al. and, finally, only the Post-CG cluster was located at 2–2.5 cm from clusters in both studies. In sum, the clusters identified via the analysis of neural adaptation effects in single letter writing were very closely aligned with many of the spelling/writing regions identified in the two meta-analyses word spelling/writing. Not surprisingly, there was some overlap between the individual studies that were entered into the meta-analyses, 5/11 studies in Purcell et al. (2011b) 5/18 in Planton et al. (2013), nonetheless, there is still a considerable amount of non-overlap, rendering quite striking the similarities observed between the results of the current study and the two meta-analyses.

It is also worth considering the similarity between the results of the current study and previous papers examining written production of single letters (Longcamp et al., 2003; James and Gauthier, 2006). However, we should first point out that both of these previous studies focused on the issue of the relationship between letter perception and production and, therefore, the experimental conditions and ROIs were selected in ways that somewhat complicate the comparison with the current study. Nonetheless, both the left fusiform and Pre-CG sites we identified are similar in location to sites reported in James and Gauthier (2006) and the SFG site we identified is similar to one of the two frontal sites reported in Longcamp et al. (2003).

In addition to evaluating the locations of the clusters identified in our study vis a vis clusters identified in the meta-analyses, we can also evaluate the extent to which the clusters identified in our study captured the full range of regions identified in the two meta-analyses. In this regard, the regions in which Purcell et al. (2011b) identified significant clusters and we did not, were: left SMA, right insula, bilateral STG/STS, IFG, and the left thalamus and putamen. Regions in which Planton et al. (2013) reported significant effects and in which we did not were: left medial frontal gyrus, right SFS, right IPL, and the thalamus and putamen.

One interpretation of these discrepancies is that they correspond to cognitive processes that are not tapped in single-letter writing. This interpretation would be consistent with the fact that the other neuroimaging studies of single letter writing, also did not report activation in these regions (Longcamp et al., 2003; James and Gauthier, 2006). One exception is the IFG which James and Gauthier (2006) reported was recruited when participants wrote or imagined the letter the first letter of the word corresponding to a seen shape (e.g., writing R when a rectangle was presented). However, in this task participants must first generate a word and its spelling and then write only the first letter. Because of this, the task includes lexical processing which may explain recruitment of this site. This interpretation of IFG recruitment in the James and Gauthier (2006) study would be consistent with the fact that both Rapp and Lipka (2011) and Rapp and Dufor (2011), using very different types of spelling tasks, found significant word frequency effects in this region, suggesting its role in lexical orthographic processing. The region has also been strongly associated with other cognitive functions such as cognitive control (Brass and Von Cramon, 2002) and lexical selection in speaking (Martin et al., 1994; Thompson-Schill, 2005). This constellation of findings prompted Rapp and Lipka (2011) to suggest that the region may play an important role in lexical selection in spelling. The fact that the single-letter writing task did not produce significant activation in this region is certainly consistent with this proposal. Another prominent region in which we did not find significant activation was the bilateral STG/STS region reported in Purcell et al. (2011b). This area has been consistently associated with processes involved in phoneme-grapheme conversion (for functional neuromaging findings see Omura et al., 2004; for lesion-based findings see Henry et al., 2007 and Philipose et al., 2007), processes that would not have been relevant to single letter production. In addition, at least some of the other non-overlapping regions, the medial frontal areas in particular, may support processes specifically required for producing multiple letters, such as processes involved in representing and monitoring the ordering and sequencing of elements. Finally, it is important to note that we cannot rule out that, at least for some of these regions, we simply lacked the power to detect effects or, alternatively that these regions may instantiate processes that are involved in letter writing but which are not susceptible to repetition habituation.

In summary, we found a striking similarity between the neural substrates identified by a neural adaptation approach based on single-letter writing and the spelling/writing meta-analyses based largely on word-based spelling/writing tasks. This provides confirmation of the utility of the approach for investigating the neural network underpinning neural adaptation spelling/writing.

### Revealing the dimensions of letter representation

Analysis 2 examined the letter-processing clusters identified in Analysis 1 for the magnitude of release from inhibition that was generated when, after repeating the same letter three times, participants produced a different letter. Specifically, this analysis evaluated if there were different degrees of release from inhibition on Change Trials depending on whether a change in letter case, shape, and/or identity was involved. As indicated in the Introduction, the significance of this analysis is that it allows for a fairly direct test of the nature of the representations processed within a region, providing a number of advantages relative to the more traditional task comparison approach.

#### Letter identity

Of the eight letter-sensitive regions identified in Analysis 1, only one exhibited a pattern of release from inhibition that indicated selective sensitivity to letter identity—the left fusiform region (TAL −41, −60, −13/MNI −43, −59, −21). Specifically, there was a significantly greater response for trials that involved a change in identity (**I**CS + **I**S) compared to those that did not (CS + C). For example, the neural response to the fourth letter in sequences such as ttt**P** and ttt**p** was greater than in sequences such as qqq**Q** or vvv**V**. That is, although all of the sequences involved a change in the shape and/or case of the letter produced, this region showed sensitivity *only* to the change in letter identity. In other words, this brain area was selectively sensitive to the abstract letter identity (ALI) that is shared by letters despite the differences in their shapes.

This interpretation is consistent with other neuroimaging findings indicating a role for this cortical area in the representation of ALIs. First, this area has not only been consistently associated with word spelling/writing, but it has also been associated with reading and, in that context, has been referred to as the Visual Word Form Area (VWFA) (Cohen et al., 2000). Furthermore, there have been several studies showing that these same substrates, in the same individuals, are active during both reading and spelling (Purcell et al., 2011a; Rapp and Dufor, 2011; Rapp and Lipka, 2011). One account of this overlap is that it occurs because reading and spelling share abstract representations of letter identities. A second line of evidence supporting the representation of ALIs in this region consists of the set of studies reporting similar neural responses or priming effects in this area for orthographic stimuli presented in different fonts (Gauthier et al., 2000; Qiao et al., 2010; Braet et al., 2012; Nestor et al., 2012) or, more importantly, priming effects that occur across case (Dehaene et al., 2001, 2004; Polk and Farah, 2002). Results such as these indicate the involvement of a representational type—such as ALIs—that abstracts across the shape differences present in letters differing only in font or case (ROSE/rose). Finally, Rothlein (Rothlein and Rapp, 2013) used an MVPA Representational Similarity Analysis approach to analyze the similarity/dissimilarity structure of neural responses generated by single-letter viewing. They were able to distinguish between representations of letter shape, letter name, and ALIs, finding evidence for the selective representation of ALI's in a region consistent with the identity-selective region identified in the current study. Against this backdrop, the contribution of the current study is that it provides additional evidence for the representation of abstract letter identities in the left mid-FG, based on evidence from single letter writing.

#### Letter shape

We identified two clusters that were responsive only to changes in letter shape: SFS and SFG/pre-CG. These two areas exhibited a larger response for trials that involved changes in shape (whether or not there was an identity change) (e.g., tttp, tttP, qqqQ) than for those involving only changes in case (e.g., vvvV). Of all the clusters identified in this study, the SFS cluster (MNI: −25, −8, 54) is the cluster that most closely aligns with the findings from the Purcell et al. and Planton et al. meta-analyses, differing only in 3–4 mm from the locations of clusters reported in these studies. It is located near the intersection of the SFS and the pre-central sulcus in a region sometimes referred to as Exner's area (Exner, 1881; Roux et al., 2009, 2010). The name originally derives from the observation of lesions resulting in writing-specific deficits and, based on the lesion literature, Exner's area is typically located in the posterior MFG, a site that is more inferior than what is more commonly observed in neuroimaging studies [see Lubrano et al. (2004), for discussion]. The shape-selectivity that we observed for this region is consistent with the finding from the Purcell et al. study that this region was identified by the subset of contrasts isolating peripheral from central writing processes and not by those isolating central spelling processes. This finding is also seen consistently in a range of individual studies designed to isolate peripheral from central components of spelling (Katanoda et al., 2001; Beeson et al., 2003; Sugihara et al., 2006; Roux et al., 2009; Rapp and Dufor, 2011; Segal and Petrides, 2012). Roux et al. (2009) suggested that this general area be referred to as the GMFA (Graphemic/Motor Frontal Area) to emphasize its role as an interface between abstract letter identities and the generation of relatively abstract, effector-independent motor commands. Rapp and Dufor (2011) reported sensitivity in this region to the length of words to be written and proposed its involvement in some aspect of orthographic WM, consistent also with the finding by Purcell et al. (2011a) that the region is recruited in both handwriting and typing. However, it is worth noting that the SFS cluster we report is also within about 1 cm from the frontal eye fields (FEF) coordinates reported by Paus (1996). This close association of FEF with a region that is consistently identified with writing has been previously noted and discussed (Matsuo et al., 2003). Previously, one concern has been that, although, participants were not viewing their writing in most of the neuroimaging studies reporting activity in this region, it might be possible that eye-movement patterns were, nonetheless, significantly different in writing vs. control conditions. The fMRI adaptation approach taken here makes this explanation somewhat less likely. We found that changes in letter shape produce greater activity in this area than changes in case alone and, while not impossible, it seems unlikely that these different conditions would yield significant differences in implicit eye-movements. Furthermore, the finding that lesions to this region specifically affect writing (Anderson et al., 1993; Tohgi et al., 1995; Cloutman et al., 2009) also makes it unlikely that the region is solely dedicated to eye-movement planning.

The other shape-sensitive cluster that we identified was located in the posterior SFG/pre-CG area (MNI −28, −20, 66), more posterior and superior to the SFS cluster discussed just above. This region has similarly been associated with motor planning, a function generally consistent with its shape sensitivity. On this basis, one possibility is that these two clusters are simply part of a larger region that is functionally homogeneous. Alternatively, although a distinction in the functions of these two nearby areas has not been previously proposed, the more posterior location of the SFG/pre-CG cluster suggests that it may play a more “downstream” role in the process of generating motor plans. Generally consistent with this upstream/downstream distinction is the fact that the more anterior SFS cluster, while exhibiting significant sensitivity only to shape, did show a trend (*p* < 0.07; Table 2) toward identity sensitivity, whereas the SFG/pre-CG did not show any such sign (*p* < 0.49; Table 2).

Having identified multiple and specific nodes within the left hemisphere frontal spelling/writing network, it will be critical for future studies to attempt to delineate their respective contributions. The transformation of abstract letter identities to muscle movements involves a number of representational levels that encode various aspects of letter shape. Thus, while these findings provide more specific and direct evidence than has previously been available regarding the representational content of these areas, this is only a first step and key questions concern the precise nature of the shape representations processed in these areas.

#### Shape + identity

Three of the letter-sensitive regions identified in Analysis 1 exhibited significant sensitivity to both shape and identity: left MFG (TAL −40, −8, 52), left postCG (TAL −52, −26, 42), and the right cerebellum (TAL 11, −53, −15). In other words, words, they exhibited significant effects both for the contrast designed to isolate identity (**I**S + **I**SC vs. SC + C) and the contrast designed to isolate shape effects (I**S** + ISC + C**S** vs. C).

The MFG cluster is generally within the region of the GFMA (Roux et al., 2009) and its location is also consistent with proposals that place Exner's Area in the posterior MFG (Lubrano et al., 2004). In addition, it is equidistant (1.5 cm) from both the SFS and the SFG/pre-CG clusters described just above. In other words, topographically it forms a part of the broader pre-motor, frontal area that has been so consistently linked to spelling, writing, and dysgraphia. The fact that letter identity is also significantly represented in this cluster may indicate either that there are subpopulations of neurons within this region that separately represent identity and shape or, alternatively that these dimensions are jointly represented. The findings of significant shape and identify effects in the posterior MFG and significant shape-only effects in the SFS, as well as significant shape effects plus a trend toward identity effects in the SFG/Pre-CG are consistent with the view of this region's role in the transformation of abstract letter identities into motor plans. The overall set of findings suggests a gradient of increasing sensitivity to shape, and decreasing sensitivity to letter identity. However, it will be critical to design future experiments to not only identify the specific nature of the shape representations in these nearby regions but also to determine if the MFG is more closely linked to the other frontal regions to which it is neuroanatomically proximal or to the post-CG and cerebellar regions that shared its sensitivity to both letter shape and identity.

The post-CG cluster is quite topographically distinct (2–2.5 cm) from the post-CG peaks reported in the Purcell et al. and Planton et al. meta-analyses, although this is primarily due to its considerably more lateral location. As Planton et al. (2013) note, activity in somatosensory areas is frequently observed in writing and other motor tasks and is often assumed to be due to tight coupling between tactile and motor processes, especially in terms of the proprioceptive feedback that plays such an important role for action (Rausch et al., 1998; Filimon et al., 2009 and see Christensen et al., 2007 for evidence of direct modulation of the somatosensory cortex by the premotor cortex). Under this view, however, it is somewhat unexpected to find significant effects of identity in this site, underscoring the fact that the specific functions instantiated in this region are not well-understood.

The recruitment of a right cerebellar region is consistent with motor control functions traditionally assumed for the cerebellum. However, as Planton et al. (2013) point out, it is not clear if there are any handwriting-specific cerebellar functions. As was the case for the post-CG cluster, it is quite interesting to find sensitivity to letter identity in the cerebellar region. This finding is consistent with the increasing evidence indicating a role for the cerebellar in a wide range of cognitive functions, including language (Stoodley and Schmahmann, 2009; Murdoch, 2010). This expansion of functions would be expected to be accompanied by evidence of higher-level and more abstract representations, such as letter identity. However, one should be cautious about over-interpreting this particular finding.

#### Absence of effects

There were two types of notable absences. First, there is the finding that no regions showed significant effects of release from inhibition based on a change in letter case (IS**C** + S**C** + **C** vs. IS). Second, there is the finding that neither the pre-CG nor the IPL clusters showed significant sensitivity to changes in letter identity, shape, or case.

With regard to the absence of case effects, it is first worth noting that case is most certainly mentally represented, as we are able to assign upper or lower case as needed in writing or typing, producing upper-case first letters for proper nouns in English and for all nouns in German. Furthermore, there have been a number of reports of selective disruption of case assignment following brain damage. These individuals produce the intended letters when writing, but cannot control the case in which the letters are produced (De Bastiani and Barry, 1989; Goodman and Caramazza, 1986; Patterson and Wing, 1989). However, it does not follow from these observations that case is necessarily represented independently of letter identity or letter shape. That is, there may be unitary representations that represent identity and case (e.g., [A-uppercase]) rather than compound representations with constituent and separable components (e.g., [A] + [uppercase]). Thus, one possibility is that we did not find case-selective regions because they do not exist. Another possibility, however, is that the methods (task, number of stimuli, fMRI, etc.) employed were not powerful enough to identify the relevant areas, particularly if case is represented by a small population of neurons.

The absence of significant identity or shape effects in the pre-CG and parietal clusters is also susceptible to lack of power or sensitivity arguments. Nonetheless, it is interesting to consider certain alternative accounts. The pre-CG cluster is situated in motor cortex, a region that may represent aspects of motor plans that are “below the grain” of the manipulations employed in this study. For example, this region could be sensitive to the number and complexity of strokes that compose a letter. If that were the case, none of the Change Trial conditions would have been expected to differ along these dimensions, thus eliminating the possibility of different degrees of release from inhibition across conditions.

Activation in various areas of parietal cortex (SPL, IPL, and IPS) are reliably observed in the Purcell et al. and Planton et al. meta-analyses (Purcell et al., 2011b; Planton et al., 2013), prompting some to posit a left parietal “writing center” (Sugihara et al., 2006). Furthermore, damage to this general region results in disruption to spelling and writing, sometimes affecting the production of letter forms or stroke sequencing (e.g., Alexander et al., 1992; Scarone et al., 2009) and suggesting the importance of this area for motor programming. In addition, there is also evidence that at least some areas within this broader region may be involved in orthographic WM. In earlier work we (Rapp and Dufor, 2011) identified SPL substrates sensitive to the number of letters in a word (while controlling for the amount of motor activity involved in writing words), a finding that is consistent with a role for this region in orthographic WM. Furthermore, lesions to this area have been reported that specifically affect orthographic WM while leaving letter production itself intact (Caramazza and Miceli, 1990). In fact, the parietal cluster identified in the current study is located 1.3 cm from the length-sensitive cluster peak reported in Rapp and Dufor (2011). However, if this region is involved in orthographic WM, one might have expected sensitivity to letter identity. While we may have simply failed to detect a letter-identity effect, it is also possible that a WM function responsible for sustaining activation over a period of time either was not engaged by this task or, if so, might not be susceptible to the selective adaptation/release from inhibition that this paradigm requires.

The question of the role of WM in the experimental task used in this investigation merits some discussion. It is certainly the case that the task used has a significant WM component given that on each trial a pair of letter forms and their associated colors must be encoded and retained to guide written responses throughout the trial. The task was designed in this way to provide a stimulus cue for letter production that does not involve either the visual form of the letter or the spoken name, in order to allow attribution of findings specifically to letter production processes (rather then the cue). Despite the role of some type of WM in this task, we don't expect the results that we have reported to be “contaminated” by the WM demands of the task as they are based on the comparison of brain responses to two conditions (4th trial of Change vs. No Change trials) that are matched in terms of WM demands. That is, participants never know whether they are in a Change or No Change trial until the fourth stimulus cue is presented and, therefore, they need to maintain the relevant information in WM for both trial types.

### Letters and words in production and perception

As indicated earlier, all eight clusters identified in Analysis 1 as comprising the network of regions recruited for single letter production were located in neuroanatomical regions also identified in meta-analyses of written word production. Considerable overlap between production of written words and letters would be expected, as the production of written words requires processing the abstract identities of single letters and their shapes, as well as the recruitment of the relevant motor plans and programs. In fact we find that the letter production network is a subset of the word production network, with the word production network recruiting additional neural substrates (left SMA, right insula, bilateral STG/STS, IFG, right SFS, right IPL, and the thalamus and putamen) which, presumably, instantiate the additional cognitive components required for word production, including orthographic lexical selection and retrieval, extensive graphemic buffering, and serial selection of letters for production, among others. In fact, identifying the overlap and non-overlap between word and letter production networks can contribute significantly to our understanding of the neural substrates of written word production.

While this investigation has focused on written production there has also been considerable interest in the literature in understanding the relationship between written production and perception (reading) at both the letter and word levels. Not only is this issue important for a better understanding of the cognitive and neural organization of the literate brain but it also has broader implications for fundamental questions regarding the relationship between perception and production, specifically as concerns the extent and nature of shared processes and neural substrates. While it is beyond the scope of this discussion to review this topic extensively, we will touch on a few points, especially as they relate to the work reported here. At the word level there has been behavioral, neuropsychological, and neuroimaging evidence of shared representations/processes for reading and spelling [for a review see Rapp and Lipka (2011)]. Recent functional neuroimaging studies have converged in identifying two regions that are active in the same individuals during reading and spelling: the left mid-fusiform/lateral occipito-temporal sulcus and the left IFJ (Purcell et al., 2011b; Rapp and Dufor, 2011; Rapp and Lipka, 2011). Further, the sensitivity of these two areas to word frequency but not word length (Rapp and Dufor, 2011) supports its role in lexical orthographic processing. With regard to orthographic WM, there has been some neuropsychological work indicating that orthographic WM processes may also be shared in reading and spelling (Tainturier and Rapp, 2003).

At the letter level, there has also been behavioral, neuropsychological, and neuroimaging evidence for shared representations/processes involved in letter perception and writing [for a review see James and Gauthier (2009)]. Among other things, this literature shows interference in letter perception by concurrent letter production, activation of common substrates in letter production and perception, as well as evidence that perceptual learning of letter shapes via motor practice (in adults and children) recruits areas also active in letter perception. A critical question for this research concerns identifying the nature of the shared representations: abstract identity, shape, motor plans, or motor programs. James and Atwood (2009) examined if the type of training used in the learning of pseudoletters in adults (writing, typing, or visual training) subsequently affected activation levels in brain areas engaged in the visual perception of the well-learned pseudoletters. They found that writing training specifically influenced activation during perception in the left posterior FG (TAL −43, −66, −12) and the left dorsal precentral gyrus (TAL −46, −8, 51) but not in (among other areas) the left middle FG. Relative to our findings, the James and Atwood (2009) precentral gyrus site is near the SFS and SFG sites (TAL −25, −5, 49 and −28, −16, 59) which we found to be selectively sensitive to letter shape and to the MFG location (TAL −40, −8, 52) that exhibited sensitivity to shape and identity. Interesting, the left middle FG site (TAL −43, −58, −10) that James and Atwood (2009) did *not* find to be to writing training, is extremely close to the fusiform site (TAL −41, −60, 13) that we found to be specifically sensitive to sensitive to letter identity representations that abstract away from letter shape. Indeed one would not expect letter identity representations to be selectively sensitive to the modality of training. This set of findings suggests that the motor/shape representations used in letter writing may be shared or interact with the shape rather than identity representations used in letter perception. Although clearly much work needs to be done in this area, these findings do underscore the point that for understanding the relationship between perception and production it is critical to distinguish amongst the various representational types involved.

## Conclusions

This study applied a novel approach to the investigation of the neural substrates of the specific dimensions of letter representation—identity, shape, and case. Although there have been a few studies that have applied repetition suppression paradigms (Gauthier et al., 2000; Gros et al., 2001) to investigate the neural substrates of letter representation, the use of an fMRI adaptation paradigm for these purposes with single letter writing is novel. The results indicate that the approach was successful not only in identifying key components of the overall network for writing letters, but also in providing more detailed information regarding the nature of the representations processed in these components. While no brain regions were shown to be sensitive to letter case, selective representation of letter identity was found in the left mid FG, the selective representation of letter shape was associated with the left SFG and SFS locations and sensitivity to both letter shape and identity were found within the right cerebellum, left post-central and middle frontal gyri. There are many things that we know when we know how to write letters, this study provides some understanding of how these different aspects of letter knowledge are neurally instantiated.

### Conflict of interest statement

The authors declare that the research was conducted in the absence of any commercial or financial relationships that could be construed as a potential conflict of interest.
